# Morphological and Hemodynamic Characteristics Associated With the Rupture of Multiple Intracranial Aneurysms

**DOI:** 10.3389/fneur.2021.811281

**Published:** 2022-01-20

**Authors:** Xiaoyu Tang, Longjiang Zhou, Lili Wen, Qi Wu, Xiaochang Leng, Jianping Xiang, Xin Zhang

**Affiliations:** ^1^Department of Neurosurgery, Jinling School of Clinical Medicine, Jinling Hospital, Nanjing Medical University, Nanjing, China; ^2^Department of Neurosurgery, The Affiliated Suzhou Hospital of Nanjing Medical University, Suzhou, China; ^3^Medical Imaging Center, The Affiliated Hosptial of Yangzhou University, Yangzhou, China; ^4^ArteryFlow Technology Co., Ltd., Hangzhou, China

**Keywords:** DSA (digital subtraction angiogram), aneurysm rupture, intracranial mirror aneurysms, morphologic parameters, hemodynamic parameters

## Abstract

**Background and Purpose:**

The objective of this study was to identify the morphological and hemodynamic factors associated with the rupture of multiple intracranial aneurysms regardless of patient-related factors and establish a statistical model for aneurysm rupture risk assessment.

**Methods:**

The digital subtraction angiography (DSA) data of 104 mirror intracranial aneurysms in 52 consecutive patients were retrospectively analyzed in this study. 21 morphological parameters and hemodynamic parameters were calculated by 3-dimensional reconstruction and computational fluid dynamics (CFD) simulation. Significant differences (*p* < 0.05) between the two groups were subsequently tested with the multivariate logistic regression to identify the independent risk factors. A prediction model was established based on the independent risk factors. The receiver operating characteristics (ROCs) were generated to estimate the prediction performance. A cohort of patients with multiple intracranial aneurysms admitted in our institute from January 2021 to October 2021 was introduced to verify the value of the model.

**Results:**

Significant differences between the ruptured and unruptured aneurysms were found in 15 out of 19 parameters. Bleb formation, neck width, and size ratio were independent factors in the multivariate logistic regression. A prediction model based on the three independent risk factors was established: Odds = −1.495 – 0.707 × _(Neckwidth)_ + 3.061 × _(Blebformation)_ + 2.1 × _(SR)_ (bleb formation: Yes = 1, No = 0). The area under the curve (AUC) value of the model was 0.901. In the validation cohort, the prediction model showed satisfying performance in assessing multiple aneurysm rupture risk with a sensitivity of 100% and specificity of 88.46%.

**Conclusion:**

Bleb formation, neck width, and size ratio were independently associated with aneurysm rupture status. The prediction model may help in identifying the aneurysm with high rupture risk.

## Introduction

Intracranial aneurysms (IAs) occur in about 2–3% of the general population (1). Subarachnoid hemorrhage (SAH) due to the ruptured IAs (RIAs) is associated with a high risk of mortality and disability [45% mortality in the first year (2) and 64% disability among the survivors (3)]. With the advancement of neuroimaging techniques, unruptured IAs (UIAs) have been increasingly detected. Given that only 1–2% of IAs are susceptible to rupture (4), rupture risk assessment of UIAs is critical for clinical decision-making. Treatment decision is mainly made on the basis of the PHASES score (8) in many medical centers. While, as to IA itself, only the size and location score were calculated, which may underestimate the rupture risk of IAs. It is of great significance to assess the rupture risk of IAs from the aspects of morphology and hemodynamics, especially for the treatment decision of multiple IAs. Several morphological parameters, such as size, irregularity in shape, aspect ratio (AR), and height/width (*H*/*W*) ratio may contribute to the rupture of IA (5). Meanwhile, hemodynamic changes also play an important role in the process of formation, development, and rupture of the aneurysm. These characteristics could help to determine which IA is prone to rupture and should be treated with priority. Intracranial mirror aneurysms, defined as aneurysms located in similar positions on bilaterally symmetrical arteries, provide an ideal model to study the growth and rupture of IAs. Using paired analysis, patient-related factors (e.g., age, gender, ethnicity, smoking, hypertension, and collagen genetics) can be neutralized by internal controls. To extensively investigate the potential predictive factors for aneurysm rupture, which will not be influenced by the characteristics of patient, we calculated and analyzed 21 morphological and hemodynamic parameters in 52 SAH patients with ruptured intracranial mirror aneurysms.

## Patients And Methods

### Patients and Three-Dimensional (3D) Model Reconstruction

The studies involving human participants were reviewed and approved by the Institutional Ethics Committee of the Jinling Hospital. The patients provided their informed consent to participate in this study. Between January 2015 and December 2020, 109 consecutive patients with intracranial mirror aneurysms were diagnosed at our institution. Among them, 40 patients without SAH were excluded. Besides, nine patients with other intracranial diseases and eight patients with incomplete clinical or image data were also excluded. The remaining 52 patients with a ruptured aneurysm and an unruptured aneurysm in the symmetric location were finally included. CT and angiographic imaging were reviewed by two skilled neuroradiologists (LW and QW, both of them have working experience more than 10 years) together to identify the hemorrhagic aneurysm. The RIAs were identified based on the definitive patterns of hemorrhaging on CT scans in cases that were treated endovascularly. Other RIAs were further confirmed during microsurgeries. These 52 pairs of aneurysms were subsequently divided into the ruptured group and the unruptured group. All the patients underwent digital subtraction angiography (DSA) examinations and 3D reconstructions were obtained from rotational angiograms using the software Syngo Workplace (Siemens, Munich, Germany), which could create a spatial reconstruction of the vasculature in STL (stereolithography) format from axial DSA images in the DICOM (digital imaging and communications in medicine) format. All these 3D models were subsequently induced into 3-Matic Medical software (version 13.0, Materialise, Leuven, Belgium); the aneurysms together with parent arteries were isolated as the region of interest. After the processes of “trim” and “local smoothing,” the models were stored for further calculation and analysis.

### Calculation of Morphological Parameters

**Figure 1:**
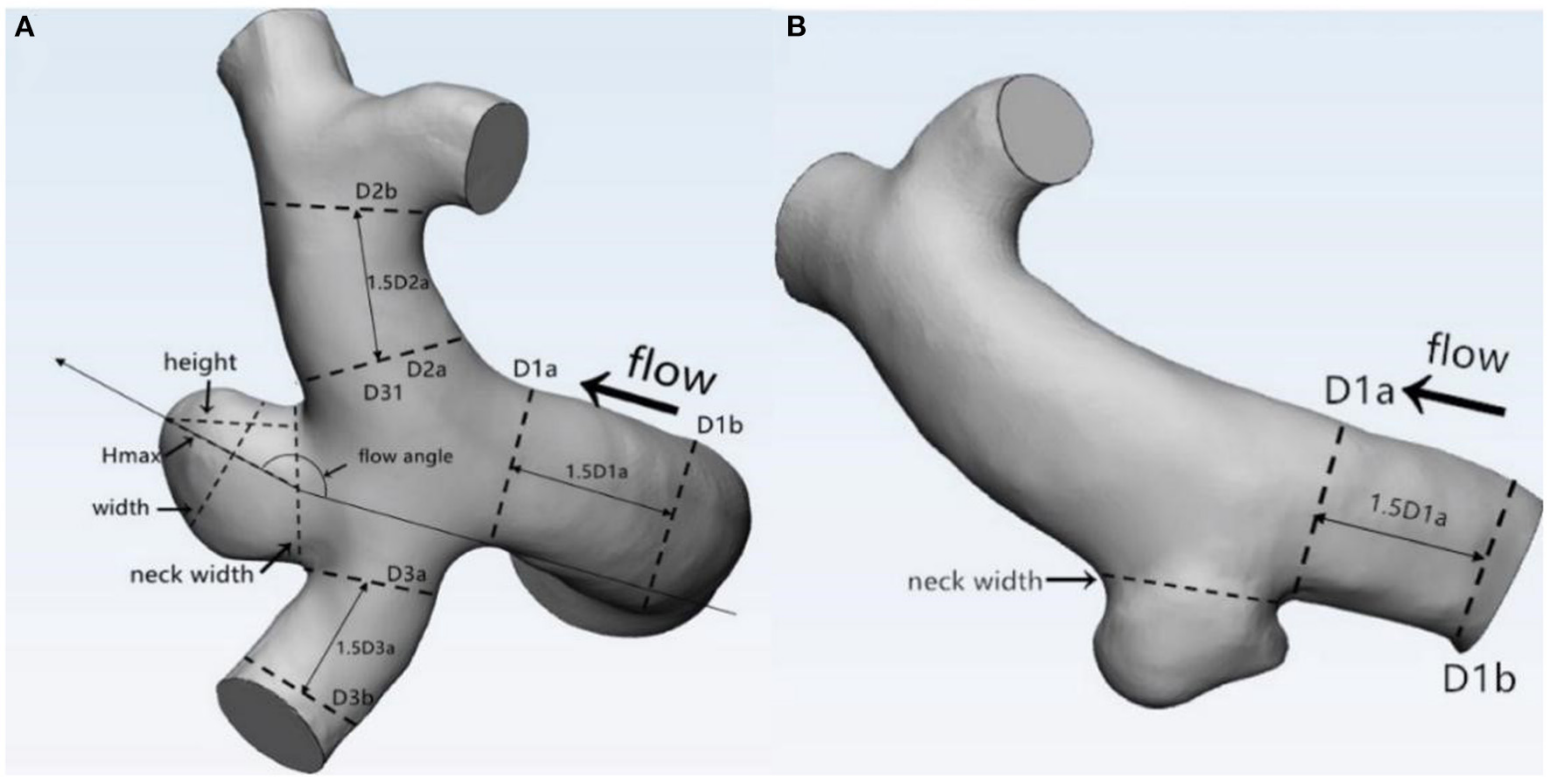
Definitions of morphological parameters (**A**: Bifurcation aneurysm, **B**: Sidewall aneurysm): Maximum size: the maximum distance of the dome from the neck plane; Neck width: the largest cross-sectional diameter of the aneurysm neck; Hmax: the maximum distance of the dome from the centroid of the neck plane; Height: the perpendicular distance of the dome from the neck plane; Width: the maximum diameter perpendicular to height; *H*/*W* ratio = height/width; bottleneck factor (BNF) = width/neck width; aspect ratio (AR) = height/neck width; size ratio (SR) = Hmax/DV, and DV were determined by averaging the diameter of the cross-section of a vessel (Da) just proximal to the neck of the aneurysm and the diameter of the cross-section (Db) at 1.5 times Da from the neck of the aneurysm. DV (mean diameters of parent vessels) = [(D1a + D1b)/2 + (D2a + D2b)/2 + (D3a + D3b)/2+ … (Dna + Dnb)/2]/n.

The morphological parameters of the aneurysms and the parent vessels were measured and independently recorded by two neuroradiologists on the workplace system. Both of these two independent observers were blind to clinical manifestations and CT results of patients. Fourteen morphological parameters, namely, irregular shape, formation of bleb, bifurcation, maximum size, neck width, Hmax, height, width, *H*/*W* ratio, Hmax/neck width, bottleneck factor (BNF), AR, size ratio (SR), and inflow angle, were calculated. Definition of: (1) irregular shape: presence of protrusions or multiple lobes (>50% of the parent aneurysm size); (2) formation of bleb: detection of the sac <50% of the parent aneurysm size; and (3) bifurcation aneurysm: aneurysm originates at bifurcation of the artery, involving at least two vessels. Other parameters were previously described by Dhar et al. (5). Parameter definition and measurement are shown in Figure 1. Average values of all these parameters were adopted for further statistical analysis.

### CFD Simulation and Calculation of Hemodynamic Parameters

Each geometry was imported into 3-Matic Medical software and meshed to create 800,000 to 1.8 million volume tetrahedral elements and wall prism elements for subsequent fluid dynamics computation. Incompressible Navier–Stokes equations were numerically solved under pulsatile flow conditions using the OpenFOAM version 6 software. The pulsatile velocity waveform obtained from transcranial Doppler ultrasound measurement on a normal individual was used as the inflow boundary condition. The flow waveforms were scaled to achieve a mean internal carotid artery (ICA) inlet flow rate of 4.6 ml/s under pulsatile conditions. The outlet was modeled as an opening boundary condition with zero static pressure and the mass flow rate through each outlet artery was proportional to the cube of its diameter (6). Blood flow was assumed to be incompressible Newtonian fluid, with a density of 1,060 kg/m^3^ and a viscosity of 0.0035 Ns/m^2^. All the models were assumed to have rigid walls with no-slip boundary conditions. We discretized the complete cardiac cycle of 1 s by a time step of 0.004 s for numeric simulation. Three pulsatile cycles were simulated to obtain stable results and the last cycle was taken as output. Hemodynamic parameters, including mean wall shear stress (WSS), mean maximum WSS (MWSS), mean parent vessel WSS (PTWSS), mean normalized WSS (NWSS), and mean oscillatory shear index (OSI) were calculated by CFD simulation. WSS is a mechanical force that acts tangential to the vessel wall.

WSS = $\frac{1}{T}\int_{0}^{T}|WSSi|dt$. [*WSSi* is the instantaneous shear stress vector and T is the duration of the cycle (6)].

Definitions of WSS-related parameters: WSS: time-averaged WSS further averaged over the dome area (the entire luminal surface of the aneurysm sac); MWSS: the maximum value of WSS during the cardiac cycle; PTWSS: WSS value acted on the parent artery wall; NWSS: mean intra-aneurysmal WSS magnitude normalized by the PTWSS.

Oscillatory shear index represents the directional change of WSS during the cardiac cycle (7).


$$\begin{array}{l} OSI=\frac{1}{2}\{1-\frac{\left|\int_{0}^{T}WSSi\;dt\;\right|}{\int_{0}^{T}\left|WSSi\right|\;dt}\} \end{array}$$

### Statistical Analysis

The quantitative data were presented as mean ± SD or median ± interquartile range. The paired *t*-test was used for normally distributed parameters, while the Wilcoxon signed-rank test was used for nonnormally distributed parameters. The qualitative data were expressed as a percentage and the McNemar's test was used to compare the differences between the two groups. The SPSS version 26 (IBM Incorporation, Armonk, New York, USA) software was used for statistical analysis. Value of *p* < 0.05 was considered to be statistically significant. When the *p*-values for variables were <0.05, they were included in the conditional, forward multiple logistic regression to calculate the independent risk factors. The receiver operating characteristic (ROC) analysis was performed, after which the area under the curve (AUC) values for each parameter were calculated and compared. Thresholds for optimal sensitivity and specificity were also calculated.

## Results

A total of 52 patients with 104 intracranial mirror aneurysms were enrolled including 41 patients with the Hunt–Hess grades I-II and 11 patients with grades III-IV. There were 11 men and 41 women with a mean age of 63.19 ± 11.00 years (range 34–86 years), of which 11 (20.37%) had an alcohol drinking habit. In terms of comorbidities, 25 (48.08%) patients had a history of hypertension and five (9.62%) patients had coronary heart disease. Five pairs of aneurysms were located in the ophthalmic segment of ICA, 31 pairs of aneurysms were posterior communicating artery aneurysms, and the left 16 pairs were mirror middle cerebral artery aneurysms. Aneurysm rupture occurred on the left side in 25 (48.08%) patients and on the right side in 27 (51.92%) patients.

### Morphological and Hemodynamic Characteristics

The values of relevant parameters are shown in Tables 1, 2. Compared with the unruptured group, the aneurysms in the ruptured group were more likely to be irregular and susceptible to blebbing and the differences were significant (*p* < 0.001). The maximum size of the aneurysms was 7.70 ± 3.30 mm in the ruptured group, which was significantly >4.42 ± 1.97 mm in the unruptured group (*p* < 0.001). The parameters including height, width, Hmax, and neck width were all found significantly greater in the ruptured group than those in the unruptured group. The AR, BNF, and SR of the ruptured aneurysms were significantly higher than the unruptured aneurysms. The inflow angle in the ruptured group was slightly larger than that in the unruptured group (*p* = 0.733). The significant variables were analyzed in the multivariate logistic regression with a conditional forward method. As shown in Table 2, neck width, formation of bleb, and SR were independently associated with the rupture status of IAs.

**Table 1:** The univariate analysis of morphological and hemodynamic characteristics associated with rupture of the aneurysm.

**Factors**	**Aneurysm**					
	**Ruptured** **(***n =*** 52)**	**Unruptured** **(***n =*** 52)**	***t***/**χ^2^**	***P*** **-value**	**AUC**	**Threshold**
**Bifurcation (%)**						
Yes	43(82.7)	38(73.1)	1.396	0.237	0.548	
No	9(17.3)	14(26.9)				
**Irregular (%)**						
Yes	40(76.9)	7(13.5)	42.275	**<0.001**	0.817	
No	12(23.1)	45(86.5)				
**Formation of bleb (%)**						
Yes	38(73.1)	5(9.6)	43.178	**<0.001**	0.817	
No	14(26.9)	47(90.3)				
Max size (mm)	7.70 ± 3.30	4.42 ± 1.97	6.149	**<0.001**	0.834	5.36
Height (mm)	5.50 ± 2.36	3.04 ± 1.53	6.293	**<0.001**	0.817	3.63
Width (mm)	5.55 ± 2.94	3.63 ± 1.85	3.958	**<0.001**	0.754	3.91
Neck width (mm)	5.96 ± 1.02	4.46 ± 1.51	2.717	**0.008**	0.633	3.67
Hmax (mm)	6.14 ± 2.65	3.36 ± 1.52	6.540	**<0.001**	0.834	4.08
Bottle neck factor	1.25 ± 0.43	0.95 ± 0.23	4.333	**<0.001**	0.694	1.10
Aspect ratio	1.25 ± 0.44	0.81 ± 0.25	6.355	**<0.001**	0.812	0.90
Size ratio	2.06 ± 0.77	1.06 ± 0.47	7.476	**<0.001**	0.873	1.31
Height/Width	1.03 ± 0.26	0.86 ± 0.24	3.794	**0.001**	0.712	0.88
Hmax/width	1.15 ± 0.28	0.96 ± 0.24	3.747	**<0.001**	0.691	0.95
Inflow angle(°)	108.18 ± 23.22	106.42 ± 28.68	0.342	0.733	0.519	100.9
WSS_mean_ (Pa)	4.71 ± 3.58	6.98 ± 5.71	2.429	**0.017**	0.315	3.875
WSS_max_ (Pa)	29.22 ± 17.23	26.96 ± 17.55	0.663	0.509	0.549	24.11
OSI_mean_	0.039 ± 0.041	0.025 ± 0.022	2.115	**0.037**	0.676	0.025
WSS_normal_	0.70 ± 0.41	0.97 ± 0.61	2.683	**0.009**	0.334	0.63
WSS_meanpt_ (Pa)	7.18 ± 4.94	7.40 ± 3.40	0.263	0.793	0.485	6.17

*Variables showing statistical significance (p < 0.05) are in bold*.

**Table 2:** The multivariate logistic regression analysis of factors associated with rupture of the aneurysm.

**Factors**	***β***	**S.E**.	**Wald**	**Sig**.	**OR (95%CI)**
Neck width	−0.707	0.283	6.250	**0.012**	0.493 (0.283, 0.858)
Bleb	3.061	0.767	15.916	**<0.001**	21.358 (4.746, 96.105)
SR	2.100	0.642	10.684	**0.001**	8.166 (2.318, 28.764)
Constant	−1.495	0.907	2.714	0.099	0.025 (0.001, 0.687)

*Variables showing statistical significance (p < 0.05) are in bold*.

**Figure 2:**
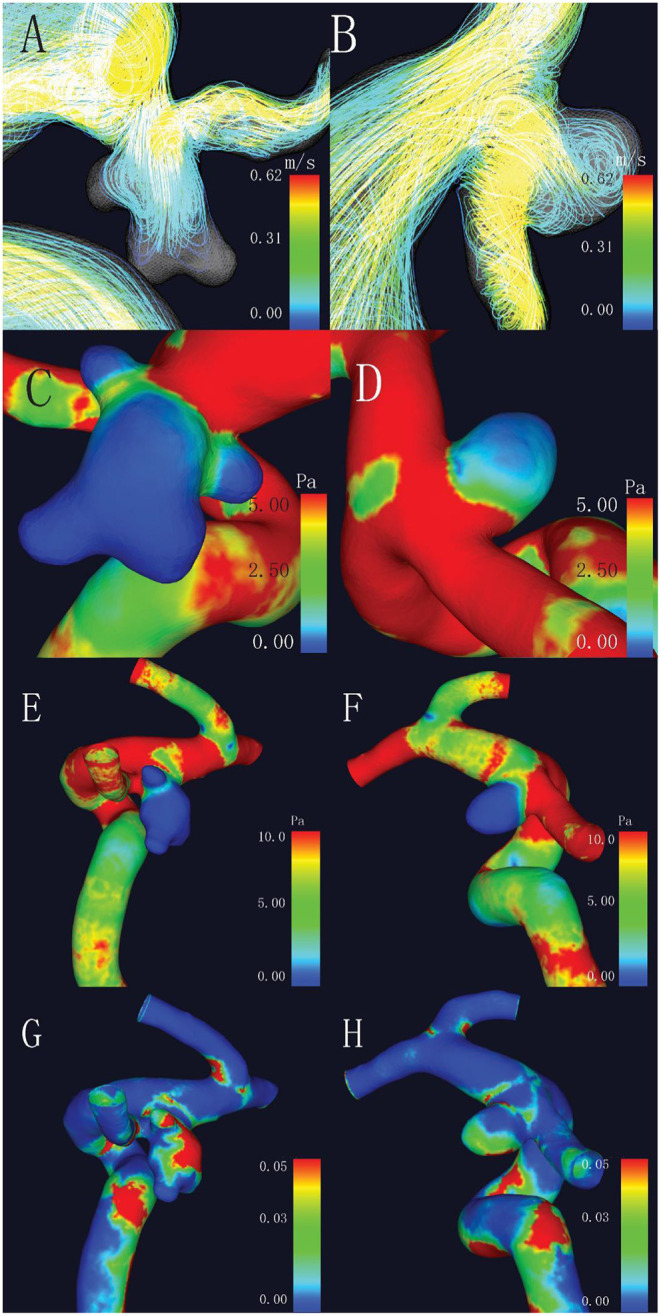
Comparison of hemodynamic characteristics of mirror aneurysms in a typical patient. **(A)** Complex flow pattern in ruptured aneurysm with blebs; **(B)** Simple flow pattern in unruptured aneurysm; **(C,D)** Lower wall shear stress (WSS) was observed in the ruptured aneurysm (2.47 vs. 2.72 Pa, *p* = 0.017); **(E,F)** No statistical differences in maximum WSS (MWSS) (44.45 vs. 14.56 Pa, *p* = 0.509); and **(G,H)** Higher oscillatory shear index (OSI) in the ruptured aneurysm (0.031 vs. 0.025, *p* = 0.037).

As for the hemodynamic parameters, CFD simulation demonstrated that ruptured aneurysms had significantly lower WSS (4.71 ± 3.58 vs. 6.98 ± 5.71 Pa, *p* = 0.017). Nevertheless, there were 13 pairs of the ruptured aneurysms with higher WSS, a higher rate of bleb formation (61.54 vs. 15.38%, *p* = 0.041), and greater size (6.11 ± 1.60 vs. 4.03 ± 2.18 mm, *p* = 0.003) were found in the subgroup. There was also a significant difference of OSI between the two groups (*p* = 0.037). Intra-aneurysmal maximum WSS and parent vessel WSS between the ruptured and unruptured aneurysms showed no statistical significance (Figure 2).

**Figure 3:**
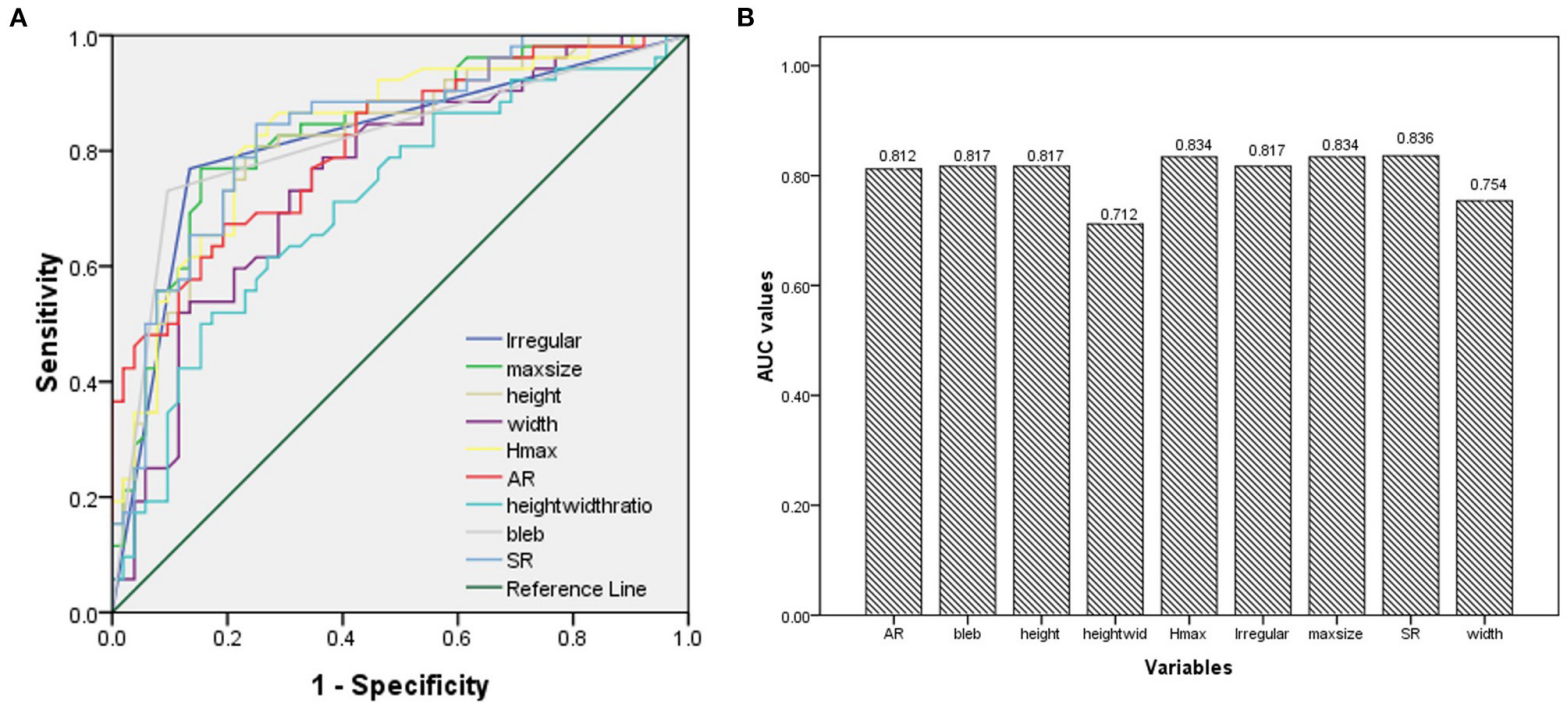
**(A)** Results of the receiver operating characteristics (ROCs); **(B)** The area under the curve (AUC) values for key parameters.

The ROC analysis and the AUC values were performed for each index in Table 1. Irregular shape, formation of bleb, maximum size, height, Hmax, AR, and SR had good AUC values (>0.8). The ROC curves for key parameters are shown in Figure 3.

**Figure 4:**
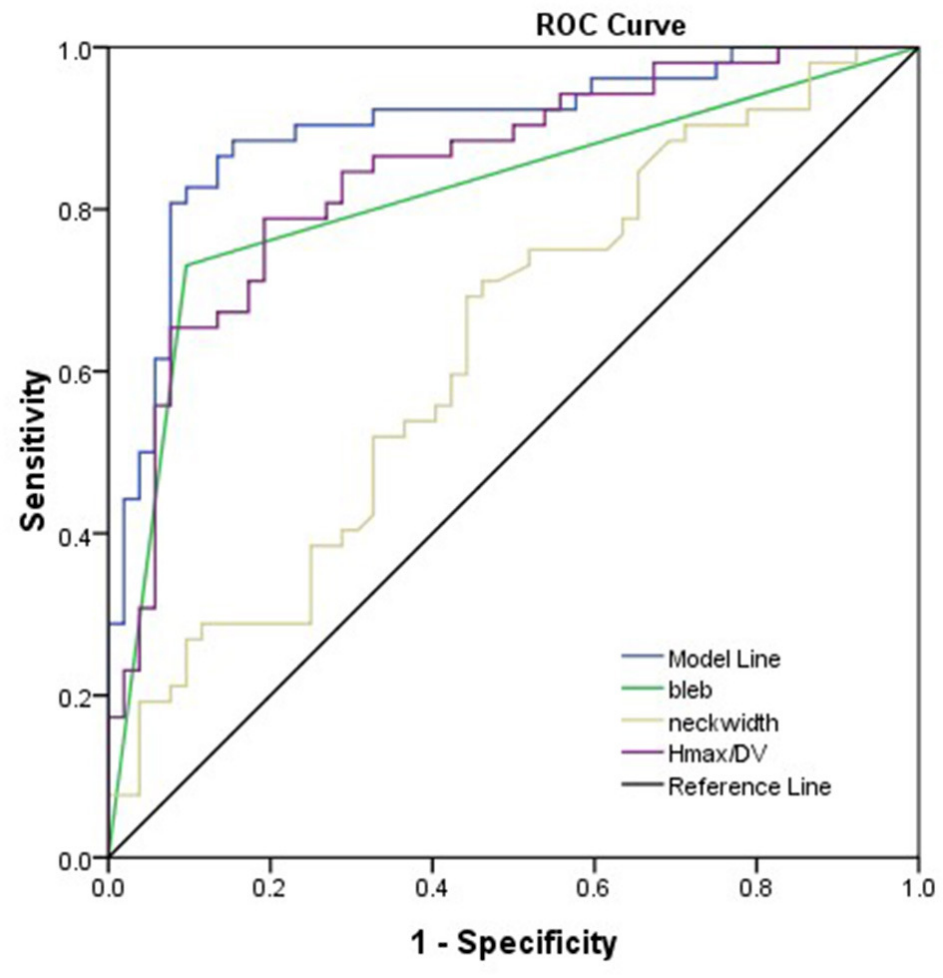
Presentation of the AUC (0.901) of the model. Bleb formation, SR, and neck width were the parameters leading to the prediction quality of models with respect to rupture status of the aneurysm.

As shown in Table 2, bleb formation, SR, and neck width were independently associated with rupture of IA. Then, a predictive model of the rupture status of IA was established: logit(*P*) = −1.495 – 0.707 × _(Neckwidth)_ + 3.061 × _(Blebformation)_ + 2.1 × _(SR)_ (bleb formation: Yes = 1, No = 0). The ROC curve was used to evaluate the predictive power of the model. The AUC value of our model was 0.901 (Figure 4). The cutoff point was −0.1016 with a sensitivity of 0.827 and specificity of 0.934. A validation cohort of 27 patients (admitted in our institute between January 2021 and October 2021) with 56 IAs was induced to verify the value of the prediction model. It showed a satisfying sensitivity of 100% (10/10) and specificity of 88.46% (46/52). The total accurate rate of recognition was 89.29% (50/56).

## Discussion

The rupture risk assessment of IAs is critical for clinical decision-making as well as the prevention of hemorrhagic stroke. According to the previous studies (8, 9), many clinical factors including age, hypertension, aneurysm location, population, and history of SAH were regarded as risk factors for aneurysm rupture. Some morphological and hemodynamic characteristics were considered as effective predictors of the rupture of IA. Thus, we combined these two aspects comprehensively, hoping to reveal the most significant features associated with the rupture of IA. We chose intracranial mirror aneurysms as study objects to avoid the internal bias induced by patient specificities. In this study, we demonstrated that formation of bleb, SR, and neck width were independently associated with the rupture of IA. Additionally, we established a prediction model for rupture risk based on the multivariate logistic regression results.

Previous study (10) has shown that bifurcation is associated with the formation and rupture of IAs. Compared with sidewall-type IAs, bifurcation-type IAs presented more complicated flow patterns in our CFD simulation, but it was not a statistically significant predictor in our series. This contradiction may be related to strict inclusion criteria of mirror aneurysms, which usually formed at the bifurcation of cerebral arteries. Lindgren et al. (11) found that irregular or multilobular shape is strongly associated with the rupture in IAs of all sizes and independent of location and patient background. Our findings also demonstrated that both of the two features are effective predictors of the rupture of IAs. Blebs are usually initiated at or adjacent to impingement regions with high WSS (12). The formation of blebs with lower WSS and higher OSI may contribute to the process of the rupture of IA, which was also observed by our CFD simulation. Since the findings were in accordance with prior conclusions, IAs with blebs should be cautiously monitored. Size of IA was identified as one of the most effective predictors in former studies. The International Study of Unruptured Intracranial Aneurysms (ISUIA) (9) found that the size and location of aneurysm are important predictors of hemorrhage and the critical size of aneurysms for rupture was 7.0 mm. In this study, however, smaller aneurysms ruptured in 3 cases (2 posterior communicating artery (PComA) aneurysms and 1 middle cerebral artery (MCA) aneurysm). Compared with the unruptured IAs, 2 ruptured smaller aneurysms presented irregular shape and the third one showed lower mean WSS in CFD simulation. Accordingly, aneurysm size is not the only consideration for management of multiple IAs; the location and shape of the aneurysm should be taken into account as well. Two previous studies (13, 14) on multiple IAs determined the cutoff sizes of 4 and 7 mm to be associated with aneurysm rupture. The threshold of maximum size for rupture was 6.15 mm in this study, which was within this range. Inflow angle was considered to be a significant discriminant of rupture status in sidewall aneurysms (15). However, in this study, inflow angle showed no significant difference. Sophisticated three-dimensional (3D) vascular structure of bifurcated aneurysms may be the underlying reason.

Five 2-dimensional parameters: AR, BNF, SR, *H*/*W*, and Hmax/Width were all proved efficient predictors in our series. Higher AR indicates more advanced phase of IA evolution. Higher volume and smaller outflow area restrict the rapid evacuation of blood flow and produce more obstruction vortexes, both of which accelerate the degeneration of endothelium, which might lead to the growth and rupture of IAs. Consistent with the study of Brian et al. (16), BNF and *H*/*W* (Hmax/width) exhibited predictive abilities in this study. Nevertheless, BNF and *H*/*W* ratio are highly dependent on aneurysm sizing methodology, which decrease the validity of the results and limit the value in prediction (17). The SR incorporates the geometry of the IA and its parent vessel. Higher SR indicates greater deformation of IAs and/or smaller diameter of the parent vessel. Given that all of these aneurysms are symmetrically located at the same segment of ICA, larger aneurysms were expected to have thinner walls. Since SR depended mainly on the change of Hmax, according to Laplace's law, larger aneurysms undergo greater wall tension when they are subjected to the same pressures. Thinner wall and greater wall tension make SR a reliable predictor. Some previous studies (18–20) also determined that SR is an important risk factor for the rupture of IA, with threshold values of 1.5, 1.8, and 1.21. SR was considered as one of the independent risk factors with a threshold value of 1.31 in this study. Another independent parameter “neck width” appeared as a protective factor of the rupture of IAs [odds ratio (OR) 0.493, *p* = 0.012], though ruptured IAs tend to present greater neck diameters. Higher flow velocity and stronger impact were observed in the narrow-necked IAs through CFD simulation, which would accelerate the damage and remodeling of the aneurysmal wall.

Many hemodynamic parameters were studied by numeral CFD simulation, among which WSS and OSI were most widely investigated. Previous studies have suggested that lower WSS is significantly correlated with the rupture status of cerebral aneurysms (20, 21). Miura Y et al. thought that significantly lower WSS may be the most reliable parameter characterizing the ruptured MCA aneurysms (21). However, some studies have shown a contrary relationship. Cebral et al. (22) found that the ruptured aneurysms have higher WSS compared with the unruptured aneurysms. Meng et al. (23) believed that both the high and low WSS can drive the growth and rupture of IA. Strong correlation between lower intra-aneurysmal WSS and aneurysm rupture was noted in this study. It should be noted that 25% (13/52) of the ruptured aneurysms in this study had higher WSS than the unruptured aneurysms. Bleb formation and/or greater size may lead to the contradictory finding. Hence, we hypothesized that morphological characteristics of surrounding vessels may contribute to the various distributions and magnitudes of intra-aneurysmal WSS. The interactions of the geometry and blood flow patterns together promote the rupture of aneurysms. Consistent with previous studies (24, 25), we observed that the low WSS region was heterogeneously distributed at the dome or the bulb regions of the aneurysm, where the aneurysm growth and rupture typically occur.

Though numerous morphological and hemodynamic characteristics were calculated and recorded simultaneously, only three morphological features showed independent predictive value. Since they could be obtained conveniently, the described prediction model can easily be assessed and used in the clinical setting. However, due to the inclusion criteria of mirror IAs, no postcirculation or anterior communicating artery (AComA) aneurysm was enrolled in the process of modeling. In the validation phase, 27 patients with 56 IAs were enrolled, among which three ruptured AComA aneurysms, four unruptured AComA aneurysms, and one unruptured basilar artery aneurysm were all detected correctly. On account of the limited number of the validation group, the accuracy of the model still needs to be verified in further investigations with larger cohort.

## Limitations

This study had some limitations. First, since mirror IAs with different status are rare in clinical practice, a limited number of patients who met the inclusion criteria were enrolled and the relatively low sample size may lead to a potential statistical bias. Second, deformation of the ruptured aneurysms may occur after bleeding and the hemodynamic parameters may be changed obviously at ruptured status (26) thus, affect the reliability of the results. Third, although some conventional parameters, such as rigid wall, laminar flow, and Newtonian blood flow, are widely accepted and applied in CFD simulation, the differences between simulation and real situation exist.

## Conclusion

We chose mirror IAs to identify the morphological and hemodynamic characteristics of ruptured IAs, which may be helpful in evaluation of rupture risk. The results of the multivariate logistic regression analysis showed that the neck width, SR, and formation of bleb were independently correlated with the rupture of the aneurysm. Then, a predictive model based on the three morphological parameters was established, with which efficient and convenient assessment could be made.

## Data Availability Statement

The original contributions presented in the study are included in the article/supplementary material, further inquiries can be directed to the corresponding author.

## Ethics Statement

The studies involving human participants were reviewed and approved by Institutional Ethics Committee of Jinling Hospital. The patients/participants provided their written informed consent to participate in this study.

## Author Contributions

XT and LZ: conceived the study and wrote the manuscript. LW and QW: collected, analyzed data, and drafted the paper. XL, JX, and XZ: revised the draft paper. All authors contributed to the article and approved the submitted version.

## Funding

The present research was supported by the National Natural Science Foundation of China (No. 82071328) and Natural Science Foundation of Jiangsu Province (No. BK20191231).

## Conflict of Interest

XL and JX are employed by company ArteryFlow Technology Co., Ltd. The remaining authors declare that the research was conducted in the absence of any commercial or financial relationships that could be construed as a potential conflict of interest.

## Publisher's Note

All claims expressed in this article are solely those of the authors and do not necessarily represent those of their affiliated organizations, or those of the publisher, the editors and the reviewers. Any product that may be evaluated in this article, or claim that may be made by its manufacturer, is not guaranteed or endorsed by the publisher.
